# Circumferential Lower Body Lift With Superficial Fascial System Preservation in Patients With BMI Below 30: A Standardized Technique and Retrospective Analysis of 110 Patients

**DOI:** 10.1093/asjof/ojag139

**Published:** 2026-06-29

**Authors:** Marcello Di Martino

## Abstract

**Background:**

Circumferential lower body lift is an effective procedure for treating truncal deformities following weight loss; however, it is associated with significant complication rates, particularly seroma formation.

**Objectives:**

The aim of this study was to describe a standardized 7-point (A-G) marking system and surgical approach for circumferential lower body lift with reduced undermining and preservation of the superficial fascial system (SFS) and to evaluate clinical outcomes and complication rates.

**Methods:**

A retrospective observational study was conducted, including 110 consecutive patients undergoing circumferential lower body lift between 2014 and 2026. Patients were stratified into bariatric vs nonbariatric and primary vs secondary procedures. Variables analyzed included age, BMI, liposuction volume, gluteal fat grafting, drain duration, and postoperative complications classified according to the Clavien–Dindo system. Statistical analysis was performed using the Mann–Whitney test for continuous variables and Fisher's exact test for categorical variables, with significance set at *P* < .05.

**Results:**

The mean BMI was 25.6 ± 2.2 kg/m^2^. The overall complication rate was 7.3%, with most complications classified as low grade according to the Clavien–Dindo system. Three seromas (2.7%) requiring outpatient aspiration were observed. Other complications included hematoma (0.9%), infection (0.9%), wound dehiscence (2.7%), and one case of deep vein thrombosis (0.9%). No statistically significant differences were found between bariatric and nonbariatric patients or between primary and secondary procedures (*P* > .05).

**Conclusions:**

Circumferential lower body lift with reduced undermining and preservation of the SFS may be a safe and effective technique in patients presenting with BMI <30 kg/m^2^. The use of a standardized 7-point (A-G) marking system may facilitate surgical planning in this selected patient population.

**Level of Evidence: 4 (Therapeutic):**

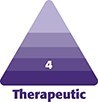

Body contouring procedures have evolved significantly over recent decades, driven by the increasing prevalence of obesity and the growing number of patients undergoing bariatric surgery.^1^ More recently, the advent of pharmacological weight-loss therapies, particularly glucagon-like peptide-1 (GLP-1) receptor agonists, has contributed to an increasing number of patients experiencing significant weight loss, thereby expanding the demand for body contouring procedures.^2^

Significant weight loss frequently results in circumferential deformities of the lower trunk, characterized by excess skin and laxity involving the abdomen, lower back, flanks, and gluteal region. These changes compromise overall body contour and are often inadequately addressed by isolated abdominal procedures. Although classically described in postbariatric patients, similar contour deformities are also observed in nonbariatric individuals, associated with aging, moderate weight loss, or decreased skin elasticity.^3-6^

Conventional abdominoplasty provides substantial improvement of the anterior abdominal contour; however, it has limited impact on the lateral trunk and lumbar region. Persistent laxity of the posterior and lateral waist remains a frequent aesthetic concern following procedures restricted to the anterior abdomen. Therefore, more comprehensive body contouring techniques have been developed to address these deformities in a global manner, simultaneously treating the anterior, lateral, and posterior trunk.^5,7^

In this context, Lockwood described the lower body lift, a procedure involving circumferential excision of skin and subcutaneous tissue combined with suspension of the superficial fascial system (SFS). This technique allows simultaneous treatment of abdominal, lumbar, and lateral trunk laxity, as well as repositioning of gluteal tissues and overall improvement of body contour.^8^ Since then, several technical variations have been described, establishing the circumferential lower body lift as one of the most effective procedures for treating circumferential deformities of the lower trunk.^7,9-11^

The association of liposuction with circumferential lower body lift has been widely adopted to optimize aesthetic outcomes. Liposuction of the dorsal region and waist allows additional contour refinement and improved waist definition, contributing to a more harmonious transition between the back, flanks, and hips.^10,12^

However, performing liposuction in conjunction with extensive skin resection requires careful preservation of anatomical structures responsible for vascularization and lymphatic drainage of the subcutaneous tissue. The SFS plays a fundamental role in vascular integrity, structural support, and lymphatic drainage.^13^ Several anatomical and clinical studies have demonstrated that preservation of this structure may contribute to maintaining tissue perfusion and reducing postoperative complications, particularly seroma formation.^14-16^

Traditional lower body lift techniques are based on circumferential en bloc resection of skin and subcutaneous tissue, often down to the muscular fascia, as described by Lockwood and later refined by authors such as Aly and Hurwitz.^5,6,8^ Although effective in correcting truncal deformities, this approach is associated with extensive tissue undermining and potential disruption of lymphatic and vascular networks. In contrast, the concept of superficial fascial preservation, particularly of Scarpa's fascia, was introduced by Saldanha in lipoabdominoplasty, demonstrating reduced complication rates, especially seroma formation.^12^ However, the systematic application of these principles to circumferential procedures—particularly in the dorsal region—remains poorly explored in the literature.

Despite the widespread use of circumferential lower body lift, most published studies focus primarily on technical descriptions or complication rates. Additionally, aspects related to standardized surgical marking—which may directly influence resection predictability, scar positioning, and tension distribution—are insufficiently systematized. Therefore, it is relevant to describe standardized technical approaches and evaluate their clinical outcomes and complication profiles in consecutive patient series.

This study aims to describe a standardized marking and surgical approach for circumferential lower body lift with reduced undermining and preservation of the SFS and to retrospectively evaluate clinical outcomes and complication rates in a consecutive patient series, including comparisons between bariatric and nonbariatric patients, as well as primary and secondary procedures.

## METHODS

### Study Design

This is a retrospective, observational study based on the analysis of a consecutive series of patients undergoing circumferential abdominoplasty or minicircumferential abdominoplasty (lower body lift) in a private clinic between October 2014 and March 2026. In this study, “minicircumferential abdominoplasty” was defined as a circumferential body contouring procedure performed in patients with a history of previous abdominoplasty who present with residual skin laxity of the flanks, waist, and posterior trunk. In these cases, a less extensive anterior skin resection is performed, typically consisting of a limited fusiform excision compared with the standard circumferential lower body lift.

The main technical difference between minicircumferential abdominoplasty and standard circumferential lower body lift lies in the extent of anterior skin resection and tissue undermining, which are more limited in the mini approach.

### Study Population

A total of 110 patients were included. Patients were stratified for comparative analysis into bariatric vs nonbariatric groups and primary vs secondary procedures.

Primary procedures were performed in patients without previous abdominoplasty, including patients with previous isolated abdominal liposuction. Secondary procedures were performed in patients with previous abdominoplasty. In these secondary cases, surgical management included either repeat abdominoplasty in patients with more significant residual abdominal laxity or miniabdominoplasty in patients with less pronounced anterior laxity, primarily aimed at correcting residual flaccidity of the flanks and posterior trunk.

### Inclusion Criteria

Patients with an indication for a circumferential lower body lift, including technical variations such as minicircumferential abdominoplasty, with complete medical records and a minimum postoperative follow-up of 8 months.

### Patient Selection and Exclusion Criteria

In our clinical practice, patients with uncontrolled diabetes mellitus, active smoking status, or BMI >30 kg/m^2^ are generally not considered ideal candidates for elective body contouring procedures until appropriate preoperative optimization is achieved. Patients not meeting these conditions underwent multidisciplinary optimization, including nutritional, metabolic, and medical management before surgical indication.

Exclusion criteria for the present study included incomplete data and early loss to follow-up. Patients with hypertension or other comorbidities were included if clinically controlled preoperatively.

This strict selection process reflects the authors’ routine clinical practice. Patients with BMI >30 kg/m^2^ are generally encouraged to continue weight reduction and achieve weight stabilization before undergoing a circumferential lower body lift. This strategy is based on the well-established association between elevated BMI and increased rates of postoperative complications, including seroma formation, wound-healing problems, infection, and venous thromboembolism. Consequently, the present study reflects outcomes obtained in a carefully selected and preoperatively optimized patient population.

### Variables Analyzed

The following variables were analyzed: age, BMI, history of bariatric surgery, weight loss, type of procedure, liposuction volume, gluteal fat grafting volume, drain duration, and postoperative complications.

### Definition of Complications

Postoperative complications recorded included seroma, hematoma, infection, wound dehiscence, thromboembolic events, and scar-related complications. Wound dehiscence was defined as a superficial separation of the skin edges not extending to the muscular fascia and not requiring operative intervention. In all cases observed in this series, dehiscence was limited to small areas of superficial wound separation and was successfully managed with local wound care, dressings, and topical antibiotic therapy. Complications were classified according to the Clavien–Dindo classification system based on the therapeutic intervention required (Supplemental Table 1).^17^

### Preoperative Marking: 7-Point (A-G) Marking System

To provide a more structured and systematic approach to preoperative planning and reduce subjectivity in surgical marking, a standardized topographic marking system based on 7 anatomical reference points (A-G) was adopted. This “7-point (A-G) marking system” provides a structured approach to defining resection lines and tension vectors in circumferential lower body lift, facilitating intraoperative consistency and surgical teaching.

**Figure 1.**
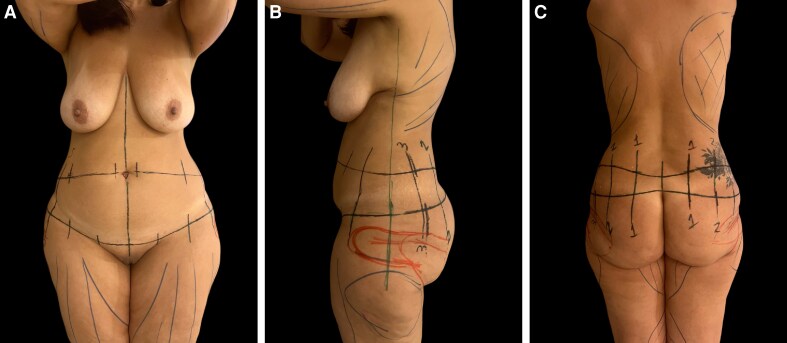
Preoperative markings: (A) anterior view, (B) lateral view, and (C) posterior view of a 48-year-old female planned for circumferential lower body lift.

Preoperative marking is performed with the patient in the standing position, allowing proper assessment of skin laxity in the abdomen, flanks, waist, and dorsal region, as well as overall body contour (Figure 1).

The abdominal midline is first marked, serving as a reference for symmetry. It should be noted that underlying musculoskeletal asymmetries, including differences in pelvic position, waist contour, gluteal projection, and trunk alignment, are relatively common. Therefore, the 7-point (A-G) marking system is used as a structured guide rather than a rigid geometric template. During marking, visual assessment is performed with the patient standing upright, and horizontal alignment is verified using anatomical landmarks and a level when necessary. The goal is to achieve the best possible aesthetic balance rather than absolute bilateral symmetry. In some cases, optimal scar positioning may require slight differences in measurements between the 2 sides in order to accommodate preexisting anatomical asymmetries.

Next, the suprapubic line is marked, corresponding to the inferior incision of the abdominoplasty. To determine its position, the patient is asked to apply upward traction to the suprapubic region, simulating the expected postoperative tension. In female patients, the line is drawn ∼6 cm above the vaginal fourchette, whereas in male patients the same principle is applied using the base of the penis as the corresponding anatomical landmark. Points A and B are subsequently defined ∼5 to 6 cm from the midline on each side, establishing Line AB as part of the 7-point (A-G) marking system.

The next step consists of identifying Points C and D in the supragluteal region, above the intergluteal cleft, using a pinch test to estimate the amount of posterior skin resection. Larger resections may be performed in patients with a short intergluteal cleft, whereas more conservative resections are preferred in patients with an elongated intergluteal cleft morphology. The position of Points C and D should be regarded as an initial estimate of the planned posterior skin excision. Before definitive resection, these markings are routinely reassessed intraoperatively. In cases where flap advancement results in excessive tension, the planned resection is reduced accordingly. This adjustment is particularly important in the supragluteal region, where excessive closure tension may increase the risk of wound dehiscence. Therefore, preservation of a tension-free closure takes precedence over strict adherence to the initial marking.

After identification of Points C and D, the posterior axillary line is identified. A second pinch test is then performed along this line to determine the degree of lateral waist skin resection, establishing Points E and F. Following the definition of these 6 reference points, Lines B-E and A-E are drawn, following a trajectory parallel or close to the inguinal fold, thereby defining the inferior abdominal resection line. The superior abdominal resection line is individualized according to the degree of abdominal skin laxity and tissue redundancy. Although initially estimated during preoperative marking, the definitive upper resection limit is determined intraoperatively following flap traction, allowing dynamic adjustment of skin excision and tension distribution.

Next, a midpoint between Points C and E is identified. Superior manual traction of the gluteal region is then performed to dynamically determine the ideal position of the supragluteal incision, establishing Point G.

The posterior inferior resection line is subsequently defined by connecting Points E-G-C, whereas the posterior superior resection line is defined by connecting Points F to D.

The aesthetic objective of the marking strategy is to position the final scar within commonly used underwear and bikini lines whenever possible. Posteriorly, the scar is intended to lie near the natural transition between the lower back and the gluteal region, while maintaining adequate tissue resection and contour improvement. Laterally, the markings are designed to provide a smooth transition between the anterior and posterior components of the excision. Anteriorly, the goal is to maintain a low suprapubic scar similar to that of a conventional aesthetic abdominoplasty. Although these represent the intended scar positions, final placement is influenced by individual anatomy and intraoperative tissue behavior.

These markings should be interpreted as an initial surgical plan rather than an exact prediction of the final scar position. The definitive extent of resection and final scar location are influenced by intraoperative tissue characteristics, flap mobility, skin elasticity, and tension distribution during closure. Therefore, the marking system serves primarily as a guide for surgical planning and is routinely refined intraoperatively according to real-time tissue behavior.

Although the initial markings are performed with the patient standing upright, all measurements are systematically reassessed intraoperatively. Before definitive skin resection, the planned excision pattern is reevaluated using flap traction and direct comparison of corresponding anatomical landmarks. When necessary, measurements are confirmed using a surgical caliper to optimize scar position and tension distribution.

**Figure 2.**
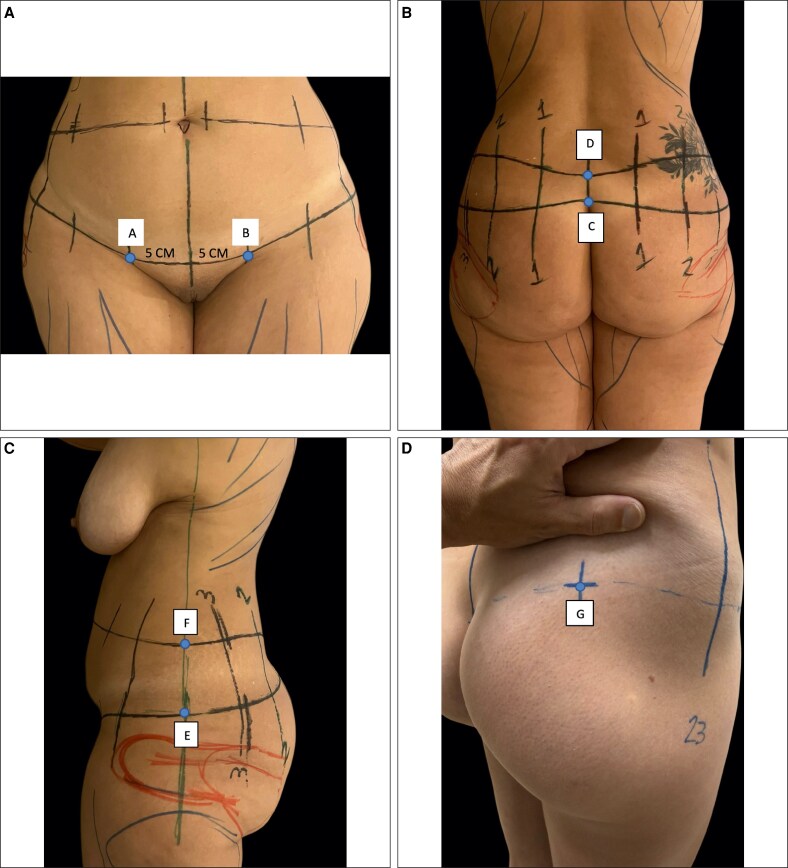
Standardized preoperative marking sequence demonstrating the 7-point (A-G) marking system in a 48-year-old female planned for circumferential lower body lift. (A) Anterior marking demonstrating Points A and B and the suprapubic reference line. (B) Posterior view showing identification of Points C and D following pinch test. (C) Lateral marking showing definition of Points E and F after pinch test. (D) Dynamic maneuver used to define Point G in a different patient.

**Figure 3.**
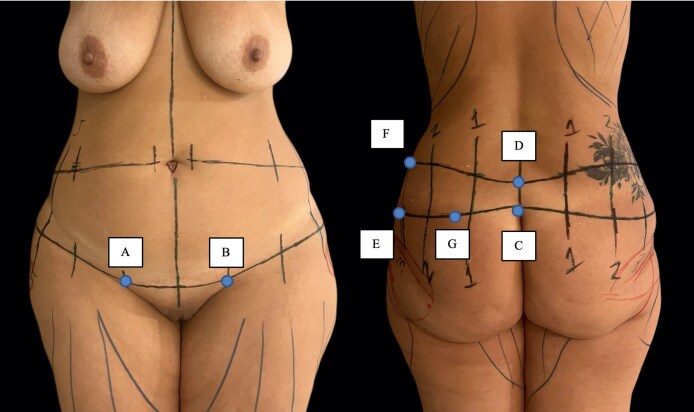
Final preoperative marking demonstrating the 7-point (A-G) marking system, including anterior (Points A and B) and posterior (Points C-G) views.

Completion of these markings establishes the final circumferential lower body lift resection pattern (Figures 2, 3).

### Surgical Technique

The patient is initially positioned in the prone position. All procedures were performed under general anesthesia. Extensive liposuction of the dorsal region, flanks, and waist is performed, including both deep and superficial planes, with preservation of the SFS.

Liposuction was performed using power-assisted liposuction associated with ultrasound-assisted emulsification (VASER; Solta Medical, Hayward, CA). A superwet infiltration technique was used. For each 2000 mL of normal saline, the infiltration solution contained 20 mL of lidocaine 2%, 20 mL of ropivacaine 7.5 mg/mL, and 4 mg of epinephrine, resulting in a final epinephrine concentration of ∼1:500,000. Total anesthetic doses were adjusted according to patient body weight and remained within accepted safety limits throughout the procedure. Liposuction was primarily performed using 3.5 mm cannulas.

In the area planned for skin excision, liposuction is performed more aggressively, involving both superficial and deep planes, in order to reduce tissue thickness and improve contour definition.

The incision is performed along the inferior line of the preoperative marking. Dissection is then carried out in a controlled manner above the SFS, using electrocautery with meticulous hemostasis.

**Figure 4.**
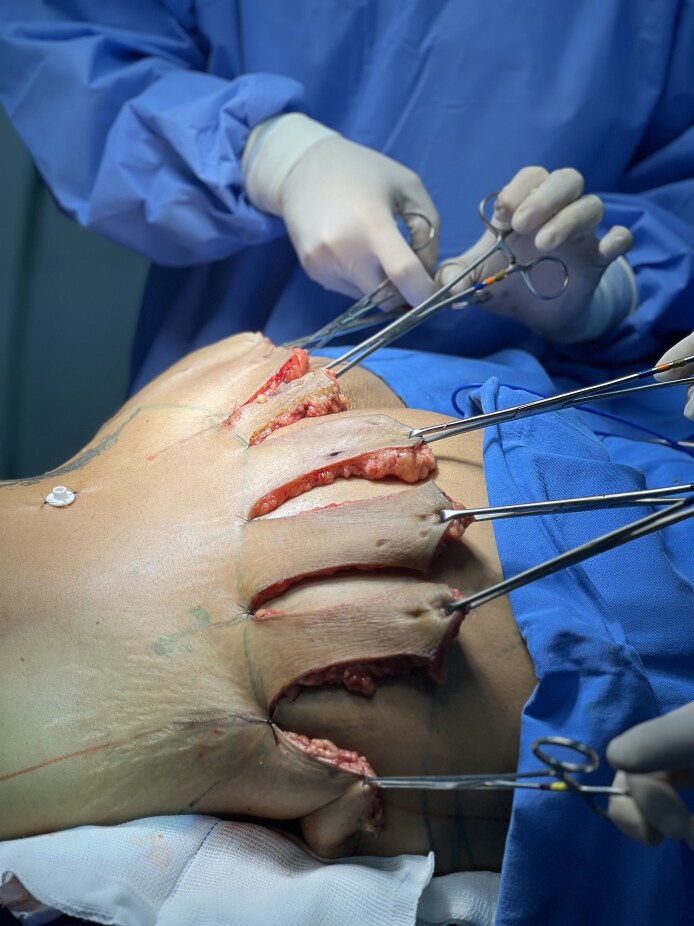
Intraoperative traction of the posterior skin flap following initial dissection above the superficial fascial system. This maneuver allows real-time reassessment of the planned resection and adjustment of the skin excision to achieve adequate contour while avoiding excessive tension on closure.

At this stage, the skin flap is manually advanced and tension is assessed intraoperatively to reassess the planned resection. This maneuver allows adjustment of the amount of tissue to be excised according to individual tissue characteristics and closure tension. The objective is to achieve adequate contour improvement while avoiding excessive tension that could compromise wound healing or increase the risk of wound-related complications. This assessment is based on intraoperative tissue behavior and surgeon experience rather than on a fixed measurement (Figure 4).

**Figure 5.**
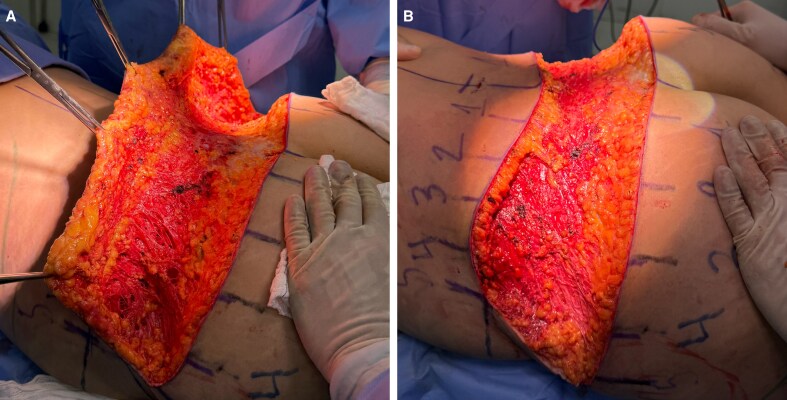
Intraoperative views. (A) Dorsal region after liposuction with preservation of the superficial fascial system, demonstrating maintained flap thickness. (B) After direct excision of excess tissue without extensive undermining.

Following this evaluation, the skin and superficial subcutaneous tissue are excised, preserving the deeper layer beneath the SFS (Figure 5).

No avulsion technique is used at any stage of the procedure.

**Figure 6.**
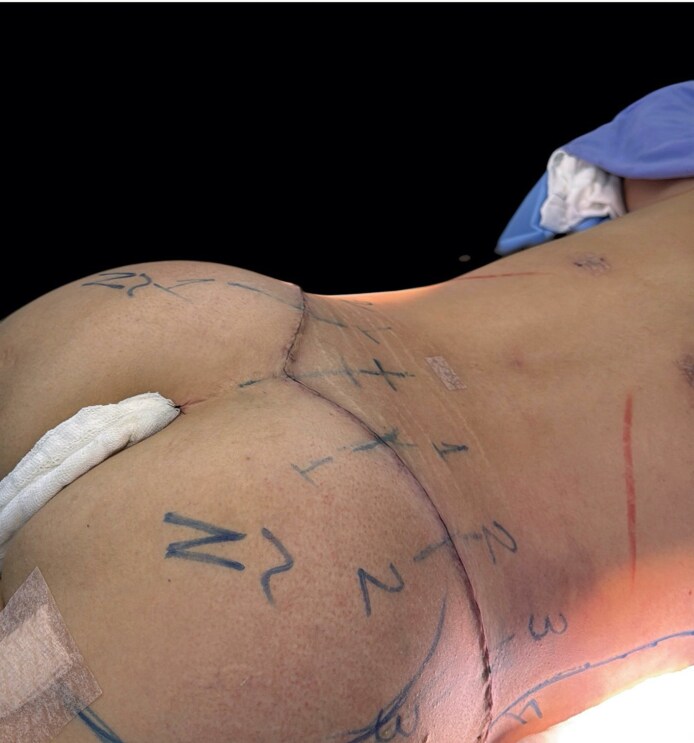
Layered closure following resection, demonstrating approximation of tissue planes and tension distribution.

After resection, progressive tension sutures are placed to reduce dead space, followed by layered closure (Figure 6). Progressive tension sutures and deep fixation sutures were placed using Nylon 2-0, followed by subdermal closure with PDS 4-0 (Ethicon, Somerville, NJ) and intradermal closure using Monocryl 4-0 (Ethicon).

When indicated, gluteal fat grafting was performed as part of posterior contour enhancement based on patient preference, preexisting gluteal volume, donor fat availability, and overall aesthetic goals. Harvested fat was decanted using standard sedimentation containers and subsequently transferred using 60 mL syringes connected to blunt-tip cannulas. Fat grafting was performed under direct visualization within the subcutaneous plane of the gluteal region and, when indicated, into the trochanteric depressions to improve lateral contour. Ultrasound guidance was not utilized.

### Anterior Stage

The patient is repositioned in the supine position. The incision is performed along the premarked line.

A conventional abdominoplasty was then performed. In contrast to the posterior stage, preservation of Scarpa's fascia was not systematically maintained throughout the anterior abdominal flap. Dissection was carried superiorly to the xiphoid process, whereas lateral undermining was kept limited to the medial third of the rectus abdominis muscles, allowing access to the line alba while minimizing unnecessary tissue undermining.

Rectus plication was systematically performed using a continuous running barbed suture (Stratafix 0, Ethicon).

No anterior abdominal liposuction was performed in any case included in this series. Liposuction was restricted to the dorsal region, waist, flanks, upper back, thighs, and other selected areas according to individual patient characteristics.

Progressive tension sutures were systematically placed throughout both the supraumbilical and infraumbilical abdominal flap using multiple fixation points spaced ∼3 cm apart, providing effective dead-space obliteration and flap stabilization.

At the end of the procedure, two 4.8 mm closed-suction drains (Portovac) were placed through a suprapubic exit sites. Each drain traversed the abdominal compartment and extended toward the posterior surgical field, allowing drainage of the extensive posterior liposuction areas and circumferential surgical dissection. This drainage strategy was adopted to avoid drain exit sites through the circumferential scar and thereby improve scar quality. Postoperative care followed a standardized protocol. Lymphatic drainage and taping were initiated ∼24 h after surgery. Compression garments were used for 60 days, often combined with foam plates.

Scar management included micropore taping during the first 30 days. From the first to the fourth postoperative month, silicone-based adhesive sheets were applied for ∼12 h during nighttime, whereas daytime care consisted of regular scar hydration using emollient topical agents.

All patients had a hospital stay of ∼24 h. Thromboembolic prophylaxis included intermittent pneumatic compression devices initiated immediately after anesthetic induction and maintained during surgery and the immediate postoperative hospitalization period. After discharge, patients were instructed to continue using elastic compression stockings for 10 days postoperatively.

Pharmacologic prophylaxis with enoxaparin was initiated 12 h after surgery and continued for 7 days, with extension up to 30 days in higher-risk patients. Patients considered at higher thromboembolic risk included those with confirmed thrombophilia or a previous history of thromboembolic events. In these cases, perioperative thromboprophylaxis protocols were individualized after hematology evaluation according to each patient's risk profile.

Antibiotic prophylaxis consisted of intravenous cefazolin administered at anesthetic induction, with routine intraoperative redosing every 4 h, followed by a 7-day postoperative course. Although current guidelines often support discontinuation of prophylactic antibiotics within 24 h after clean surgical procedures, our institutional protocol includes a short postoperative course because a circumferential lower body lift combines extensive circumferential soft-tissue dissection, prolonged operative times, large wound surfaces, associated liposuction, and temporary drain use. For these reasons, postoperative antibiotics have traditionally been maintained as part of our perioperative management protocol.

### Statistical Analysis

Comparisons were performed using the Mann–Whitney test for continuous variables and Fisher's exact test for categorical variables. A significance level of *P* < .05 was adopted.

This study was conducted in accordance with the principles of the Declaration of Helsinki. All data were retrospectively collected and fully anonymized. All patients had previously signed informed consent authorizing the use of clinical data and photographic records for scientific publication and educational purposes. Because of the retrospective design and the absence of identifiable patient information, formal ethics committee approval was not required in accordance with local regulations.

## RESULTS

A total of 110 patients undergoing circumferential abdominoplasty or minicircumferential abdominoplasty associated with liposuction between 2014 and 2026 were analyzed. The mean age was 42.3 ± 8.7 years. Of the 110 patients included, 103 (93.6%) were women and 7 (6.4%) were men. Twenty-five patients (22.7%) were bariatric, and 85 (77.3%) were nonbariatric. Regarding the type of procedure, 90 cases (81.8%) were classified as primary and 20 (18.2%) as secondary.

The mean BMI was 25.6 ± 2.2 kg/m^2^ (range, 21.8-29.6 kg/m^2^). The mean liposuction volume was 2034.5 ± 817.5 mL, whereas the mean gluteal fat grafting volume was 288.5 ± 83.1 mL per side. Gluteal fat grafting was performed in 109 patients (99.1%). The mean drain duration was 7.1 ± 1.7 days. The mean postoperative follow-up duration was 12.1 ± 4.6 months, ranging from 8 to 36 months. Demographic and surgical characteristics are presented in Table 1.

**Table 1.** Demographic and Surgical Characteristics of Patients (*n* = 110)

Variable	Total (*n* = 110)
Age (years), mean ± SD	42.3 ± 8.7
BMI (kg/m^2^), mean ± SD	25.6 ± 2.2
Bariatric patients, *n* (%)	25 (22.7%)
Nonbariatric patients, *n* (%)	85 (77.3%)
Primary procedures, *n* (%)	90 (81.8%)
Secondary procedures, *n* (%)	20 (18.2%)
Liposuction volume (mL), mean ± SD	2034.5 ± 817.5
Gluteal fat grafting (mL per side), mean ± SD	288.5 ± 83.1
Drain duration (days), mean ± SD	7.1 ± 1.7
Follow-up duration (months)	12.1 ± 4.6

SD, standard deviation.

**Figure 7.**
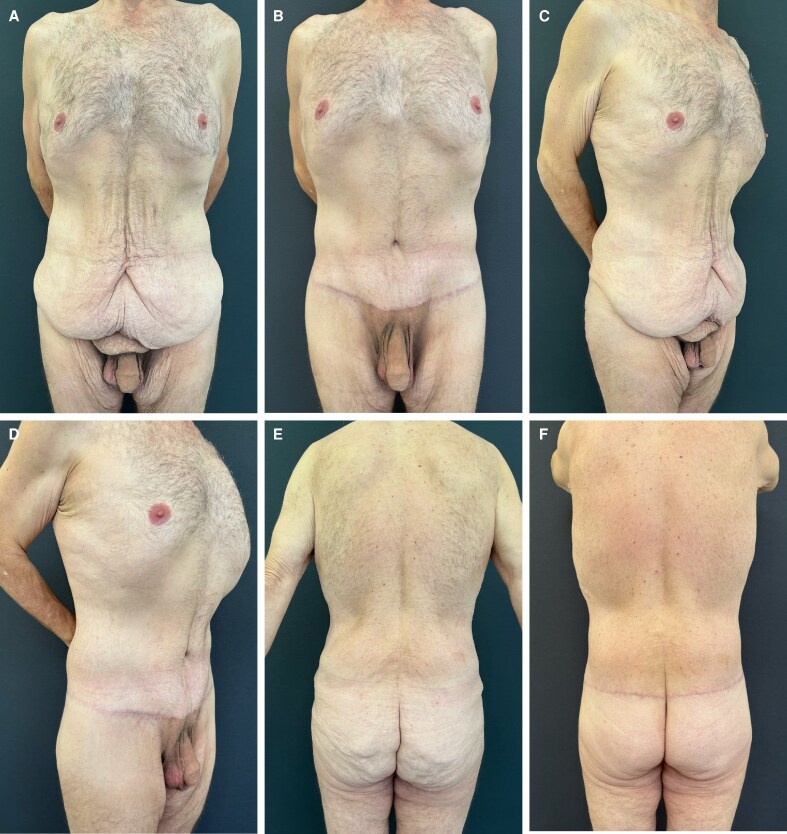
(A, C, E) Preoperative and (B, D, F) postoperative views of a 53-year-old male patient following massive weight loss who underwent circumferential lower body lift. Postoperative images obtained at 1-year follow-up demonstrate marked improvement in abdominal contour, waist definition, and gluteal ptosis, with restoration of posterior body contour.

**Figure 8.**
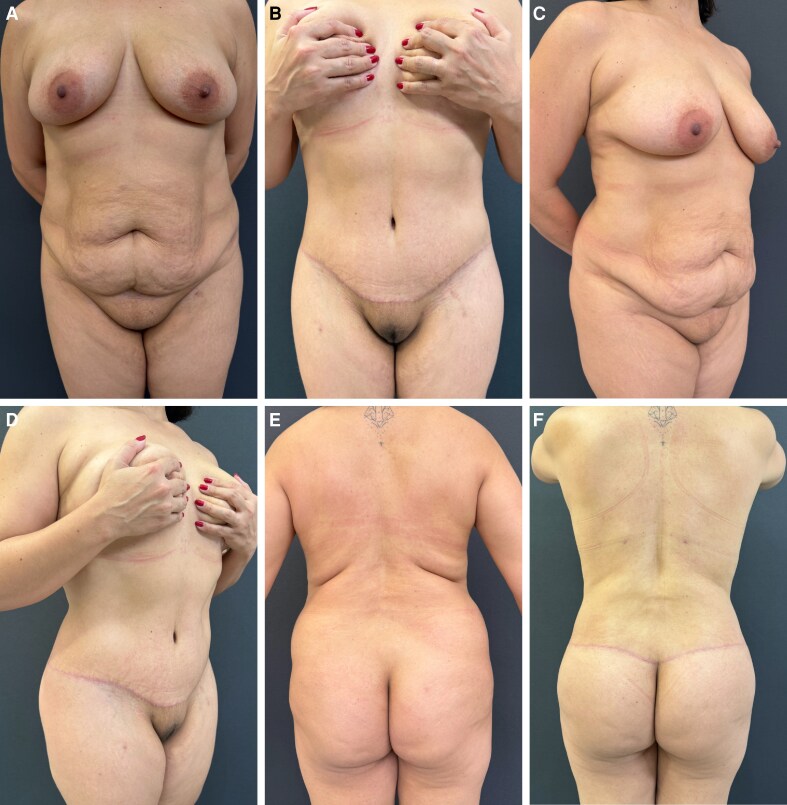
(A, C, E) Preoperative and (B, D, F) postoperative views of a 43-year-old female patient undergoing a circumferential lower body lift. Postoperative images obtained at the 8-month follow-up demonstrate improvement in trunk contour, waist definition, and posterior body aesthetics.

Representative preoperative and postoperative results are shown in Figures 7 and 8, demonstrating consistent improvement in abdominal contour, waist definition, and gluteal projection across different patient profiles.

### Group Analysis

#### Bariatric vs Nonbariatric Patients

Compared with nonbariatric patients, bariatric patients were younger (39.0 ± 8.3 vs 43.2 ± 8.9 years; *P* = .042), had greater weight loss (47.2 ± 16.3 vs 10.5 ± 12.4 kg; *P* < .001), lower liposuction volumes (1706 ± 610 vs 2131 ± 839 mL; *P* = .017), and longer drain duration (7.9 ± 1.3 vs 6.8 ± 1.7 days; *P* = .004). No significant differences were observed regarding BMI, gluteal fat grafting volume, or postoperative complication rates between groups (*P* > .05). Data are presented in Table 2.

**Table 2.** Comparison Between Bariatric and Nonbariatric Patients

Variable	Bariatric (*n* = 25)	Nonbariatric (*n* = 85)	*P*-value
Age (years)	39.0 ± 8.3	43.2 ± 8.9	.042*
Weight loss (kg)	47.2 ± 16.3	10.5 ± 12.4	<.001*
BMI (kg/m^2^)	25.4 ± 1.8	25.6 ± 2.3	.720
Liposuction volume (mL)	1706 ± 610	2131 ± 839	.017*
Gluteal fat grafting (mL per side)	264 ± 68	296 ± 86	.095
Drain duration (days)	7.9 ± 1.3	6.8 ± 1.7	.004*
Complication rate, *n* (%)	4 (16.0%)	14 (16.5%)	1.000

*Statistically significant (*P* < .05).

#### Primary vs Secondary Procedures

Of the 110 patients included, 90 patients (81.8%) underwent primary full abdominoplasty, whereas 20 patients (18.2%) underwent secondary miniabdominoplasty. Compared with secondary procedures, primary procedures demonstrated higher BMI (25.8 ± 2.1 vs 24.5 ± 2.4 kg/m^2^; *P* = .035), greater liposuction volumes (2201 ± 759 vs 1285 ± 585 mL; *P* < .001), greater gluteal fat grafting volumes (303 ± 78 vs 222 ± 74 mL per side; *P* < .001), and longer drain duration (7.5 ± 1.4 vs 5.0 ± 1.2 days; *P* < .001). No significant differences were observed in age, weight loss, or postoperative complication rates between groups (*P* > .05). Data are presented in Table 3.

**Table 3.** Comparison Between Primary and Secondary Procedures

Variable	Primary (*n* = 90)	Secondary (*n* = 20)	*P*-value
Age (years)	42.1 ± 9.0	43.4 ± 8.7	.480
Weight loss (kg)	19.6 ± 20.2	15.8 ± 21.6	.285
BMI (kg/m^2^)	25.8 ± 2.1	24.5 ± 2.4	.035*
Liposuction volume (mL)	2201 ± 759	1285 ± 585	<.001*
Gluteal fat grafting (mL per side)	303 ± 78	222 ± 74	<.001*
Drain duration (days)	7.5 ± 1.4	5.0 ± 1.2	<.001*
Complication rate, *n* (%)	16 (17.8%)	2 (10.0%)	.519

*Statistically significant (*P* < .05).

#### Complications

The overall rate of major complications was 7.3% (*n* = 8). Three seromas (2.7%) requiring outpatient aspiration were classified as Clavien–Dindo Grade IIIa complications. One infection (0.9%) treated with oral antibiotics and one case of deep vein thrombosis (0.9%) requiring anticoagulation were classified as Grade II complications. One hematoma (0.9%) and 3 cases of wound dehiscence (2.7%) managed conservatively were classified as Grade I complications.

Scar-related complications occurred in 10 patients (9.1%), including hypertrophic scars and scar widening. Most postoperative complications observed in this series were classified as low grade according to the Clavien–Dindo classification system. The full distribution of complications is presented in Table 4.

**Table 4.** Postoperative Complications According to the Clavien–Dindo Classification System

Complication	*n* (%)	Clavien–Dindo Grade
Seroma	3 (2.7)	IIIa
Hematoma	1 (0.9)	I
Infection	1 (0.9)	II
Wound dehiscence	3 (2.7)	I
Deep vein thrombosis	1 (0.9)	II
Scar complications	10 (9.1)	I

Complications were graded according to the Clavien–Dindo classification system based on the therapeutic intervention required. Individual complications are presented separately and are not cumulative.

No statistically significant differences were observed in complication rates between bariatric and nonbariatric patients, nor between primary and secondary procedures (*P* > .05), suggesting comparable complication profiles between these groups.

## DISCUSSION

Circumferential lower body lift remains one of the most comprehensive procedures in body contouring surgery, particularly in patients following significant weight loss. Since its initial description by Lockwood, several technical modifications have been proposed to improve aesthetic outcomes and reduce complication rates.^8,13^

Traditionally, the literature has focused on postbariatric patients, who are often associated with higher complication rates. In the present study, a predominance of nonbariatric patients was observed, reflecting a contemporary trend toward expanding indications of the procedure to patients with skin laxity because of aging or moderate weight loss. Despite this, no significant differences in complication rates were observed between bariatric and nonbariatric patients, suggesting that proper patient selection may mitigate traditionally reported risks.^3-6,18^

Previous studies evaluating body contouring surgery after massive weight loss have demonstrated the influence of factors such as BMI, extent of tissue resection, selective undermining techniques, and perioperative thromboembolic risk stratification on postoperative complication profiles.^18-21^

Additionally, most postoperative complications observed in this series were classified as low grade according to the Clavien–Dindo classification system, reinforcing the relatively low morbidity profile associated with the technique and careful patient selection routinely adopted in elective body contouring surgery.

The most relevant finding of this series was the low seroma rate (2.7%). Historically, seroma has been considered one of the most common complications following abdominoplasty and circumferential body contouring procedures, with older series frequently reporting rates between 15% and 35%.^4-8^ However, more recent evidence suggests a progressive reduction in seroma incidence over time, likely associated with refinements in surgical technique and postoperative management. In a 2021 systematic review and meta-analysis, Salari et al reported a global seroma prevalence of 10.9% after abdominoplasty and demonstrated a declining trend in more contemporary studies.^22^ The authors attributed this reduction to the adoption of preventive strategies such as progressive tension sutures, preservation of fascial structures, lipoabdominoplasty concepts, and more conservative dissection techniques. In this context, the low seroma rate observed in the present series may similarly reflect the combined effect of SFS preservation, reduced undermining, aggressive dead-space control, and standardized postoperative management.

The pathophysiology of seroma formation involves lymphatic disruption, dead-space formation, and lack of adherence between tissue planes. In this context, preservation of the SFS, as described by Lockwood, combined with reduced undermining and systematic use of progressive tension sutures, has been consistently associated with reduced seroma formation.^11-16^

Additionally, the composition of the study population may have influenced the outcomes. The mean BMI in this series was 25.6 kg/m^2^, lower than that reported in most classical lower body lift series, which often include patients with mean BMI between 29 and 33 kg/m^2^.^4,5,18^

Several studies have demonstrated an association between higher BMI and increased complication rates, including seroma, infection, and wound dehiscence.^3,4,22^ Patients with higher BMI present increased adipose flap thickness, greater potential for dead-space formation, and alterations in lymphatic drainage, factors that favor the development of these complications.^5,6,8,12^

In our practice, patients presenting with BMI above 30 kg/m^2^ are not immediately scheduled for surgery. Instead, they undergo a period of preoperative optimization guided by a multidisciplinary team, including nutritionists, physicians specialized in metabolic care, and physical trainers.

This process, which may extend from 6 to 12 months, aims to achieve weight stabilization and improved metabolic conditions before surgery. As a result, patients are operated on in a more favorable physiological state, which may contribute to the lower complication rates observed in this series.

Therefore, the favorable outcomes reported in this study should be interpreted within the context of a carefully selected population with BMI <30 kg/m^2^. These results may not be directly generalizable to patients with higher BMI values, who frequently present greater tissue thickness, increased dead-space formation, different marking requirements, and a higher baseline risk of postoperative complications.

Thus, the favorable outcomes observed in this study likely reflect the combined effect of a standardized surgical technique and careful patient selection. The association of reduced undermining, preservation of the SFS, systematic use of adhesion sutures, and a lower BMI profile likely played a significant role in reducing morbidity, particularly seroma formation.^7,23-25^

Although the preoperative markings may suggest a relatively conservative skin excision, the final extent of resection is determined intraoperatively. After dissection, controlled traction of the posterior skin flap allows dynamic reassessment of the resection area, enabling adjustment of skin removal while maintaining a tension-free closure.

This intraoperative refinement is particularly relevant in patients with significant weight loss, in whom aggressive resections may increase the risk of wound-related complications. Similar considerations regarding tissue tension, wound healing, and complication risk in massive weight loss patients have also been emphasized in previous lower body lift and abdominoplasty series.^26,27^

By tailoring the resection based on real-time tissue behavior, it is possible to balance contour improvement with preservation of tissue perfusion and reduced complication rates. For this reason, the proposed marking system should not be interpreted as a method capable of precisely predicting final scar position in every patient. Rather, it provides a structured framework that facilitates planning while preserving the flexibility required to accommodate individual anatomical variation and intraoperative findings.

The clinical outcomes observed in this series further support the reproducibility of the technique and its effectiveness in improving body contour.

The present study contributes to the literature by demonstrating that the standardization of surgical marking—specifically through the use of a structured 7-point (A-G) marking system—associated with preservation of the SFS may directly impact clinical outcomes in circumferential body contouring procedures. The use of this system may have contributed to a more structured approach to surgical planning, more consistent tension distribution, and possibly to the low complication rates observed in this series. To our knowledge, few studies have systematically applied SFS preservation to the dorsal region in circumferential procedures. The extension of Saldanha's principles, originally described in lipoabdominoplasty, to the posterior trunk may represent a relevant technical advancement in body contouring surgery.^12^ This approach may contribute to improved lymphatic drainage preservation and tissue vascularization, which are key factors in the pathophysiology of seroma formation, and may help explain the lower rates observed in this series.^11-16,28^ Other complications also showed low incidence rates, comparable to or lower than those reported in the literature. The rates of hematoma (0.9%), infection (0.9%), and dehiscence (2.7%) fall within or below commonly reported values.^5-8^ The incidence of deep vein thrombosis (0.9%) was also consistent with contemporary series, reinforcing the importance of appropriate prophylactic protocols.^15,19^

Regarding the analysis between primary and secondary procedures, higher liposuction volumes and longer drain duration were observed in primary cases, without a corresponding increase in complication rates. This finding suggests that the extent of the procedure alone is not the main determinant of morbidity and that surgical technique and tissue management are more relevant factors.^7,9^

Scar-related changes were observed in 9.1% of patients, a rate consistent with the literature, and likely more related to individual patient factors than to the surgical technique itself.^3,20^

This study has limitations inherent to its retrospective design, including potential selection bias and lack of randomization. However, the analysis of a consecutive series, combined with technical standardization, provides clinical relevance to the findings. An important limitation of the proposed marking strategy is that, although the 7-point (A-G) marking system provides a structured framework for surgical planning, it should not be interpreted as a universally applicable template. Individual anatomical variations, differences in tissue redundancy, musculoskeletal asymmetries, and intraoperative tissue behavior frequently require adjustments to the initial markings. Therefore, the marking strategy described herein should be viewed as a practical guide based on the authors’ experience rather than a rigid protocol applicable to all patients.

Additional limitations include the absence of validated patient-reported outcome measures and the lack of independent aesthetic evaluation. Furthermore, this represents a single-surgeon retrospective series without a formal control group, which limits the ability to directly compare outcomes with alternative techniques or to establish definitive causal relationships between specific technical aspects and complication rates. In addition, subgroup analyses were performed using groups of unequal size, particularly in the bariatric vs nonbariatric and primary vs secondary procedure comparisons, which may have reduced the statistical power to detect potential differences between groups. Future prospective multicenter studies incorporating objective aesthetic assessments and patient-reported outcomes may further validate the reproducibility and clinical impact of the proposed technique.

From a practical standpoint, the results suggest that circumferential abdominoplasty can be performed with low complication rates when fundamental principles are respected, including appropriate patient selection, preservation of anatomical structures, and the use of techniques that minimize dead space.

Additionally, the use of a standardized marking strategy, such as the 7-point (A-G) marking system, may facilitate surgical planning and teaching, reducing variability and improving intraoperative consistency among surgeons.

## CONCLUSIONS

Circumferential lower body lift and minicircumferential lower body lift with reduced undermining, preservation of the SFS, and systematic use of progressive tension sutures were associated with low postoperative complication rates in this consecutive retrospective series of patients presenting with BMI <30 kg/m^2^.

The low incidence of seroma observed suggests that the combination of limited undermining, preservation of the SFS, dead-space control, and careful patient selection may contribute to reduced morbidity in extensive body contouring procedures. The use of a standardized 7-point (A-G) marking system may facilitate surgical planning and intraoperative consistency in selected patients undergoing circumferential lower body lift. Further prospective comparative studies are necessary to validate the broader clinical applicability of the proposed technique.

## Supplemental Material

This article contains supplemental material located online at https://doi.org/10.1093/asjof/ojag139.

## Supplementary Material

ojag139_Supplementary_Data

## References

[1] Nguyen NT, Nguyen XM, Lane J, Wang P. Relationship between obesity and diabetes in a US adult population: findings from the National Health and Nutrition Examination Survey, 1999-2006. Obes Surg. 2011;21:351–355. doi: 10.1007/s11695-010-0335-4 21128002 PMC3040808

[2] Wilding JPH, Batterham RL, Calanna S, et al Once-weekly semaglutide in adults with overweight or obesity. N Engl J Med. 2021;384:989–1002. doi: 10.1056/NEJMoa2032183 33567185

[3] Song AY, Jean RD, Hurwitz DJ, Fernstrom MH, Scott JA, Rubin JP. A classification of contour deformities after bariatric weight loss: the Pittsburgh Rating Scale. Plast Reconstr Surg. 2005;116:1535–1546. doi: 10.1097/01.prs.0000182606.92069.13 16217505

[4] Shermak MA, Bluebond-Langner R, Chang D. Maintenance of weight loss after body contouring surgery for massive weight loss. Plast Reconstr Surg. 2008;121:2114–2119. doi: 10.1097/PRS.0b013e3181708129 18520903

[5] Aly AS, Cram AE, Heddens C. Truncal body contouring surgery in the massive weight loss patient. Clin Plast Surg. 2004;31:611–624. doi: 10.1016/j.cps.2004.04.004 15363914

[6] Hurwitz DJ. Single-staged total body lift after massive weight loss. Ann Plast Surg. 2004;52:435–441. doi: 10.1097/01.sap.0000123361.14654.a5 15096919

[7] Di Martino M, Nahas FX, Barbosa MVJ, et al Seroma in lipoabdominoplasty and abdominoplasty: a comparative study using ultrasound. Plast Reconstr Surg. 2010;126:1742–1751. doi: 10.1097/PRS.0b013e3181efa6c5 20639797

[8] Lockwood T. Lower body lift with superficial fascial system suspension. Plast Reconstr Surg. 1993;92:1112–1122. doi: 10.1097/00006534-199311000-00018 8234509

[9] Aly AS, Cram AE, Chao M, Pang J, McKeon M. Belt lipectomy for circumferential truncal excess: the University of Iowa experience. Plast Reconstr Surg. 2003;111:398–413. doi: 10.1097/00006534-200301000-00072 12496613

[10] Aly AS, Mueller M. Circumferential truncal contouring: the belt lipectomy. Clin Plast Surg. 2014;41:765–774. doi: 10.1016/j.cps.2014.06.008 25283461

[11] Baroudi R, Ferreira CA. Seroma: how to avoid it and how to treat it. Aesthetic Surg J. 1998;18:439–441. doi: 10.1016/S1090-820X(98)70073-1 19328174

[12] Saldanha OR, Pinto EB, Matos WN, Jr, Lucon RL, Magalhães F, Bello EM. Lipoabdominoplasty without undermining. Aesthetic Surg J. 2001;21:518–526. doi: 10.1067/maj.2001.121243 19331937

[13] Lockwood T. Superficial fascial system (SFS) of the trunk and extremities: a new concept. Plast Reconstr Surg. 1991;87:1009–1018. doi: 10.1097/00006534-199106000-00001 2034721

[14] Pollock T, Pollock H. Progressive tension sutures in abdominoplasty. Clin Plast Surg. 2004;31:583–589. doi: 10.1016/j.cps.2004.03.015 15363911

[15] Nahas FX, Ferreira LM, Ghelfond C. Does quilting suture prevent seroma in abdominoplasty? Plast Reconstr Surg. 2007;119:1060–1066. doi: 10.1097/01.prs.0000242493.11655.68 17312514

[16] Li M, Wang K. Efficacy of progressive tension sutures without drains in reducing seroma rates of abdominoplasty: a systematic review and meta-analysis. Aesthetic Plast Surg. 2021;45:581–588. doi: 10.1007/s00266-020-01913-w 32856104

[17] Dindo D, Demartines N, Clavien PA. Classification of surgical complications. Ann Surg. 2004;240:205–213. doi: 10.1097/01.sla.0000133083.54934.ae 15273542 PMC1360123

[18] Neaman KC, Hansen JE. Analysis of complications from abdominoplasty: a review of 206 cases at a university hospital. Ann Plast Surg. 2007;58:292–298. doi: 10.1097/01.sap.0000239806.43438.54 17471135

[19] Pannucci CJ, Bailey SH, Dreszer G, et al Validation of the Caprini risk assessment model in plastic and reconstructive surgery patients. J Am Coll Surg. 2011;212:105–112. doi: 10.1016/j.jamcollsurg.2010.08.018 21093314 PMC3052944

[20] Brito IM, Meireles R, Baltazar J, Brandão C, Sanches F, Freire-Santos MJ. Abdominoplasty and patient safety: the impact of body mass index and bariatric surgery on complications profile. Aesthetic Plast Surg. 2020;44:1615–1624. doi: 10.1007/s00266-020-01725-y 32342171

[21] Kolker AR, Lampert JA. Maximizing aesthetics and safety in circumferential-incision lower body lift with selective undermining and liposuction. Ann Plast Surg. 2009;62:544–548. doi: 10.1097/SAP.0b013e31819fb34a 19387158

[22] Salari N, Fatahi B, Bartina Y, et al The global prevalence of seroma after abdominoplasty: a systematic review and meta-analysis. Aesthetic Plast Surg. 2021;45:2821–2836. doi: 10.1007/s00266-021-02365-6 34080041

[23] van der Sluis N, van Dongen JA, Caris FLS, Wehrens KME, Carrara M, van der Lei B. Does scarpa's fascia preservation in abdominoplasty reduce seroma? A systematic review. Aesthetic Surg J. 2023;43:NP502–NP512. doi: 10.1093/asj/sjad024 PMC10264224 36747469

[24] Xiao X, Ye L. Efficacy and safety of scarpa fascia preservation during abdominoplasty: a systematic review and meta-analysis. Aesthetic Plast Surg. 2017;41:585–590. doi: 10.1007/s00266-017-0784-4 28405750

[25] Camargo CP, Kasmirski JA, Valente MSVS, Secanho MS, Cintra W, Gemperli R. Therapeutical strategies to prevent abdominoplasty complications: a systematic review. Aesthetic Plast Surg. 2025;49:1396–1407. doi: 10.1007/s00266-024-04563-4 39681686

[26] Losco L, Roxo AC, Roxo CW, et al Lower body lift after bariatric surgery: 323 consecutive cases over 10-year experience. Aesthetic Plast Surg. 2020;44:421–432. doi: 10.1007/s00266-019-01543-x 31748908

[27] Marchica P, Costa AL, Brambilla T, et al Retrospective analysis of predictive factors for complications in abdominoplasty in massive weight loss patients. Aesthetic Plast Surg. 2023;47:1447–1458. doi: 10.1007/s00266-022-03235-5 36609741

[28] Di Martino M, Nahas FX, Kimura AK, Sallum N, Ferreira LM. Natural evolution of seroma in abdominoplasty. Plast Reconstr Surg. 2015;135:691e–698e. doi: 10.1097/PRS.0000000000001122 25811581

