# Supplementary material for: Circumferential Lower Body Lift With Superficial Fascial System Preservation in Patients With BMI Below 30: A Standardized Technique and Retrospective Analysis of 110 Patients
Source: Aesthet Surg J Open Forum. 2026 Jun 29;8:ojag139. doi: 10.1093/asjof/ojag139 (PMC13457913; doi:10.1093/asjof/ojag139)
Supplement: Supplementary file 1 [file ojag139_Supplementary_Data.docx]

**Supplementary Table S1. Clavien-Dindo Classification of Surgical Complications**

| **Grade** | **Definition** |
| --- | --- |
| Grade I | Any deviation from the normal postoperative course not requiring pharmacological treatment or surgical, endoscopic, or radiological intervention. |
| Grade II | Complications requiring pharmacological treatment beyond those permitted for Grade I, including antibiotics, blood transfusions, or total parenteral nutrition. |
| Grade IIIa | Complications requiring surgical, endoscopic, or radiological intervention not performed under general anesthesia. |
| Grade IIIb | Complications requiring surgical, endoscopic, or radiological intervention under general anesthesia. |
| Grade IVa | Life-threatening complication requiring intensive care unit (ICU) management with single-organ dysfunction. |
| Grade IVb | Life-threatening complication requiring ICU management with multi-organ dysfunction. |
| Grade V | Death of the patient. |

Abbreviation: ICU, intensive care unit.

Source: Dindo D, Demartines N, Clavien PA. Classification of surgical complications: a new proposal with evaluation in a cohort of 6,336 patients and results of a survey. Ann Surg. 2004;240(2):205-213.
